# Altered miRNA Expression Profiles in the Serum of Beagle Dogs Experimentally Infected with *Toxocara canis*

**DOI:** 10.3390/ani13020299

**Published:** 2023-01-15

**Authors:** Wen-Bin Zheng, Lang Cai, Yang Zou, Wen-Wei Gao, Qing Liu, Xing-Quan Zhu

**Affiliations:** 1 Laboratory of Parasitic Diseases, College of Veterinary Medicine, Shanxi Agricultural University, Jinzhong 030801, China; 2 State Key Laboratory of Veterinary Etiological Biology, Key Laboratory of Veterinary Parasitology of Gansu Province, Lanzhou Veterinary Research Institute, Chinese Academy of Agricultural Sciences, Lanzhou 730046, China; 3 Key Laboratory of Veterinary Public Health of Higher Education of Yunnan Province, College of Veterinary Medicine, Yunnan Agricultural University, Kunming 650201, China

**Keywords:** *Toxocara canis*, toxocariasis, miRNA, serum, interaction

## Abstract

**Simple Summary:**

Toxocariasis is a parasitic zoonosis distributed all over the world, but is excessively neglected. Therefore, research on toxocariasis is urgently needed. MicroRNAs (miRNAs) are demonstrated to be useful biomarkers and have multiple biological functions in numerous diseases. However, research on the altered miRNA expression profiles in Beagle dogs infected with *Toxocara canis* is still inadequate, especially in the serum. Using RNA sequencing technology, the miRNA expression in the serum of Beagle dogs infected with *T. canis* was examined at 24 h, 10 days and 36 days, and several differently expressed miRNAs were identified. This study indicated that *T. canis* infection can alter the expression of miRNAs in the serum of Beagle dogs and provided baseline data to further clarify the regulatory signaling networks involved in the pathogenesis of toxocariasis.

**Abstract:**

*Toxocara canis* is a neglected roundworm, which can cause debilitating disease in dogs and humans worldwide. Serum is an excellent material for monitoring the occurrence of many diseases. However, no information is available on the expression of microRNAs (miRNAs) in the serum of dogs infected with *T. canis*. In this study, RNA-seq analysis was performed to identify the serum miRNA profiles in Beagle dogs infected with *T. canis* at different stages of infection. A total of 3, 25 and 25 differently expressed miRNAs (DEmiRNAs) were identified in dog serum at 24 h post-infection (hpi), 10 days post-infection (dpi) and 36 dpi, respectively, such as cfa-let-7g, cfa-miR-16, cfa-miR-92b, cfa-miR-93, cfa-miR-122, cfa-miR-485 and cfa-miR-451. Kyoto Encyclopedia of Genes and Genomes (KEGG) pathway analysis revealed that these miRNAs could regulate the pathways related to parasitic infectious diseases and immune system, such as amoebiasis, toxoplasmosis, platelet activation, IL-17 signaling pathway and chemokine signaling pathway. These results provide a foundation to explore the underlying regulatory role of miRNAs in definitive hosts after *T. canis* infection.

## 1. Introduction

*Toxocara* is a common zoonotic roundworm in both developing and developed countries, which is the causative agent of toxocariasis [1,2]. The average prevalence of *Toxocara* infection was 19% in humans and 11.1% in dogs worldwide [3,4]. Despite its ubiquity, toxocariasis has been neglected for decades [1]. Toxocariasis is considered as one of the six neglected parasitic diseases by the Centers for Disease Control and Prevention, and has been targeted for public health action (https://www.cdc.gov/parasites/npi/, accessed on 11 January 2023). *Toxocara canis* was identified as the main causative agent of toxocariasis, and infected humans present different clinical symptoms, some more overt, such as visceral/ocular larva migrans, while others more cryptic, such as neurotoxocariasis [5]. Canines are definitive hosts for *T. canis*; humans, many other vertebrates (e.g., rodents, artiodactyla, aves and reptiles) and invertebrates (earthworms, and cockroaches) are accidental/paratenic hosts for *T. canis* [6]. Adult *Toxocara* residing in the intestinal tract of a definitive host can excrete a large number of eggs, leading to a 21% prevalence of *Toxocara* eggs in soil samples in public places worldwide [7]. Therefore, it is necessary to consider how to reduce transmission risk and implement protocols to break the transmission cycle. Clinicians, researchers and public health officials are committed to understanding infection prevalence in definitive and paratenic hosts, and to elucidate the transmission dynamics of *T. canis* infection among these hosts [8]; however, based on the high prevalence of this parasite in humans and definitive hosts (dogs), as well as the intimate relationship of the human–dog bond, more studies on *T. canis* are still urgent. However, so far, the molecular biology of *T. canis* and the interaction mechanisms of *T. canis*-hosts remain to be clarified.

Fortunately, “omics” techniques and bioinformatics analysis have been employed to reveal the molecular biology and regulatory mechanisms of *Toxocara* [9]. The data of draft genome and transcriptome of *T. canis* provided many known protein coding genes that participate in the migration and development of *Toxocara*, and predicted the functions of unknown genes by comparing them with other relevant species [10]. The transcriptomics has been used to identify the expression profiles of messenger RNAs (mRNAs), microRNAs (miRNAs) and long non-coding RNAs (lncRNAs) in the lung of dogs infected by *T. canis*, which indicated that the pathogenesis of *T. canis* in the lung of definitive hosts is induced by both pro- and anti-inflammatory reactions [11]. The metabolomics has been used to identify the altered steroid hormone biosynthesis pathway in the serum of dogs infected by *T. canis*, which revealed the dual role of immunometabolism in the pathogenesis of toxocariasis [12].

miRNAs are short non-coding oligonucleotides typically 21~23 nucleotides (nt) in length with important roles in post-transcriptional gene regulation [13], which can regulate the expression of proteins through targeting mRNA degradation [14]. miRNAs can affect the functions of various immune cells of the host, such as leukomonocytes, monocytes and neutrophils, regulating the transcription of cytokines and intracellular signaling pathways [15,16]. Serum is an excellent material for monitoring the occurrence of diseases, and circulating miRNAs in serum have the potential to be non-invasive biomarkers for disease diagnosis, such as in *Schistosoma japonicum* and *Fasciola gigantica* infections [17,18]. The miRNAs of *T. canis* have been identified, providing valuable information for investigating the developmental biology of *T. canis* at the molecular level, as well as a potential basis for future exploration of the interactions between *T. canis* and the host [19]. However, no information is available on the expression of miRNAs in the serum of dogs infected by *T. canis*.

In this study, we analyzed the global miRNA expression profiles in the serum of Beagle dogs infected by *T. canis* at different stages using RNA-seq. The differently expressed miRNAs (DEmiRNAs) responsive to *T. canis* infection were identified and the roles of these DEmiRNAs were discussed. These results provided a foundation to explore the underlying regulatory role of miRNAs in definitive hosts after *T. canis* infection.

## 2. Materials and Methods

### 2.1. Ethics Approval

The animal experiment involving the use of Beagle dogs was reviewed and approved by the Animal Ethics Committee of Lanzhou Veterinary Research Institute, Chinese Academy of Agricultural Sciences (Approval No. 2018-015). The Beagle dogs used in this study were handled in accordance with good animal practice as stipulated by the Animal Ethics Procedures and Guidelines of the People’s Republic of China. All efforts were made to minimize the number of animals and maintain their welfare during the experiment.

### 2.2. Animals and Collection of Samples

A total of 18 serum samples of Beagle dogs were collected from the same cohort used in a previous study [12]. In brief, three litters of Beagle puppies with six per litter (n = 18), 6–7 weeks old, were divided into three groups corresponding to the process of *T. canis* development in definitive host: 24 h post-infection (hpi) (three infected puppies vs. three control puppies), 10 days post-infection (dpi) (three infected puppies vs. three control puppies) and 36 dpi (three infected puppies vs. three control puppies). Six puppies per litter were allocated randomly into the infected group and control group to reduce background differences. Puppies in the infected groups were infected orally with 1 mL of saline solution containing 300 infective *T. canis* eggs, while puppies in the control groups were mock-infected orally with 1 mL saline solution only. At each of the above indicated time points post infection, the blood of jugular vein of each puppy was collected for separating serum. Then, serum was divided into 1.5 mL tubes and stored at −80 °C for RNA-seq.

### 2.3. RNA Extraction and RNA-seq Analysis

Total RNA was isolated from each puppy serum sample at 24 hpi, 10 dpi and 36 dpi using TRIzol reagent (Invitrogen, Carlsbad, CA, USA) and miRNaesy Serum/Plasma Kit (Qiagen, Hilden, Germany) following the manufacturer’s instructions. The concentration and RIN/RQN (RNA integrity number/RNA Quality Number) of the extracted RNA were evaluated using an Agilent Bioanalyzer 2100 system (Agilent Technologies, Santa Clara, CA, USA). Ten ng of RNA per sample was used for preparing the small RNA libraries using a BGISEQ-500 small RNA UMI library construction kit (BGI, Shenzhen, China) according to a previous study [20]. Subsequently, the small libraries were sequenced using a BGISEQ-500 sequencer (BGI, Shenzhen, China). Base-calling was performed using BGISEQ-500 software (v. 0.3.8.111, BGI, Shenzhen, China).

Clean reads were obtained by removing the reads that contained the sequence of adaptor, high content of unknown bases (N) and low-quality reads. Subsequently, clean reads were mapped to the reference genome of *Canis familiaris* downloaded from the Ensembl database (release 99) by Bowtie [21]. Small RNA sequences were quantified by comparing with *Canis familiaris* small RNA mirBase database (release 21) using mirDeep2 software [22]. The read number of per known miRNAs and predicted novel small RNAs were counted by miRDeep2. pl script according to previous descriptions [20,22]. The transcription levels of miRNAs were normalized and assessed by transcripts per million of total aligned miRNA reads (TPM). DEmiRNAs between infected puppies and control puppies were assessed with a threshold of |log2 (fold change)| ≥ 1 and a *p*-value < 0.05 using *t*-test.

### 2.4. KEGG Pathway Analysis of Differently Expressed miRNAs

To better understand the potential roles of the DEmiRNAs in the serum of puppies infected by *T. canis*, the target genes of these DEmiRNAs were predicted by miRanda [23] and RNAhybrid [24]. Predicted target genes that overlapped with the two databases were selected for further analysis. The fold change of miRNA transcription was used to represent the difference in the expression of the predicted target genes, and then we summed the total miRNA fold change related to the Kyoto Encyclopedia of Genes and Genomes (KEGG) pathways. KEGG pathway analyses of the predicted target genes of DEmiRNAs were carried out using KOBAS 3.0 with the criteria of *p*-value < 0.05 [25].

### 2.5. Quantitative RT-PCR Validation of RNA-seq Results

According to the number of DEmiRNAs identified in each infection stage, 1/6 to 1/3 DEmiRNAs were randomly selected at each infection stage, and a total of 10 DEmiRNAs were selected for validating the RNA-Seq results using quantitative real-time PCR (qRT-PCR) on a LightCycler480 (Roche, Basel, Switzerland). At each infection stage, serum RNAs extracted from three infected puppies and three control puppies were used to analyze the expression level of DEmiRNA using qRT-PCR in the different groups. Commercial kits, amplification conditions and melting curve analysis for miRNAs amplification were performed as described previously [11]. Briefly, the validation was performed using a qRT-PCR kit (TianGen, Beijing, China). The amplification procedure was initial denaturation (95 °C, 15 min) and 45 cycles (94 °C, 20 s and 60 °C, 34 s). The melting curve analysis was 95 °C (10 s), 65 °C (1 min), and then gradually increased from 65 to 95 °C. The transcription levels were normalized to that of the U6 snRNA internal reference gene. All primers are shown in Table 1. The changes of expression level were calculated using the 2^−ΔΔCT^ method.

## 3. Results

### 3.1. Overview of RNA-seq Results

**Figure 1:**
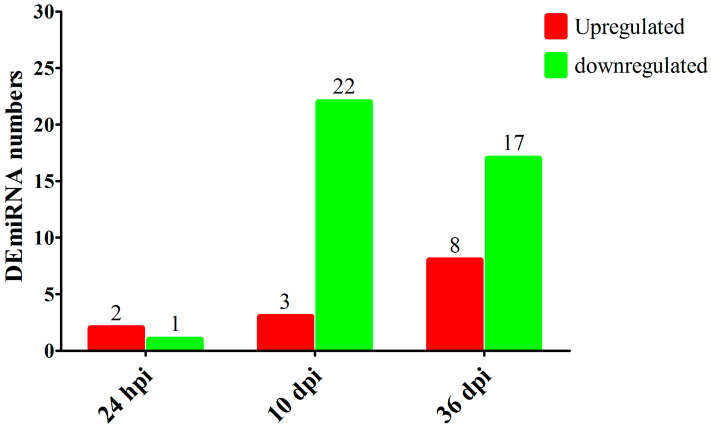
The numbers of differently expressed miRNAs (DEmiRNAs) in the serum of Beagle dogs infected by *Toxocara canis* at 24 hpi, 10 dpi and 36 dpi. The red and green colors represent the upregulated and downregulated miRNAs, respectively.

**Figure 2:**
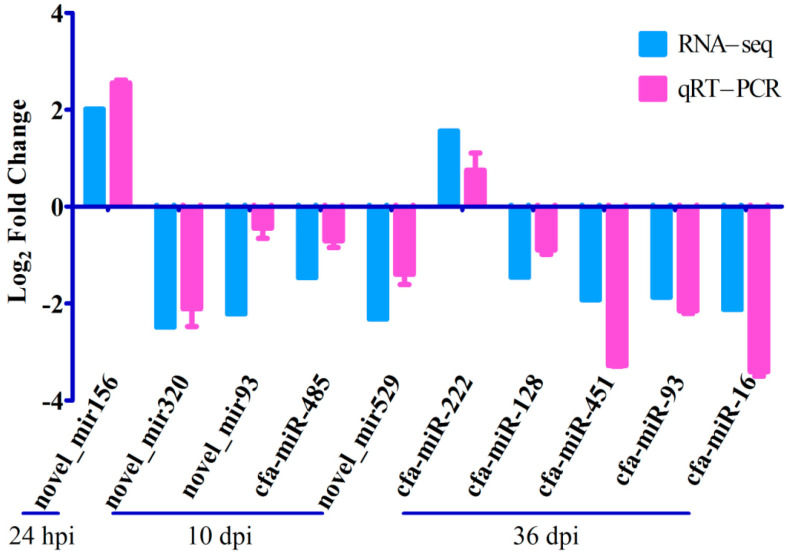
Verification of the expression of miRNAs at 24 hpi, 10 dpi and 36 dpi. The Y-axis denotes the log_2_ (fold change) and the X-axis represents differently expressed miRNAs (DEmiRNAs). The error bars represent the standard deviation based on 3 replicates.

A total of 675,893,357 raw tags and 634,967,544 clean tags were generated from 18 RNA libraries in this study. After mapping the clean tags to the genome of *Canis familiaris*, 241 known miRNAs were identified in this study (Table S1); among which, cfa-miR-92a, cfa-miR-486, cfa-miR-191, cfa-miR-451 and cfa-miR-25 were highly abundant miRNAs. Moreover, 530 miRNAs were predicted as putative novel miRNAs (Table S1); among which, novel_mir335, novel_mir196 and novel_mir484 were highly abundant miRNAs. Three DEmiRNAs were identified at 24 hpi with 2 upregulated miRNAs and 1 downregulated miRNA; 25 DEmiRNAs were identified at 10 dpi with 3 upregulated miRNAs and 22 downregulated miRNAs; 25 DEmiRNAs were identified at 36 dpi with 8 upregulated miRNAs and 17 downregulated miRNAs (Figure 1 and Table S2). The RNA-seq results were validated using qRT-PCR analysis (Figure 2), which showed that the overall trend of RNA-seq expression levels of the 10 DEmiRNAs obtained from qRT-PCR results were consistent with the RNA-seq results, confirming the reliability of the RNA-seq results.

### 3.2. KEGG Pathway Analysis of Differently Expressed miRNAs

**Figure 3:**
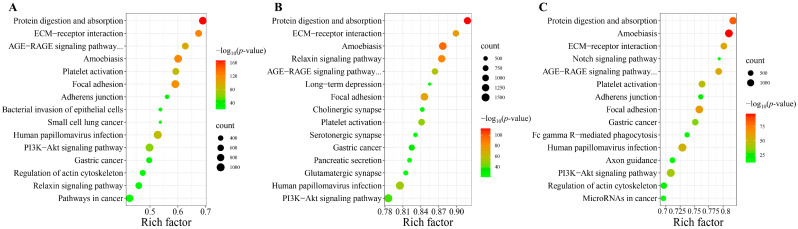
Scatter plots of the top 15 KEGG pathways of the potential targeted genes of differentially expressed miRNAs (DEmiRNAs) at (**A**) 24 h post-infection (hpi), (**B**) 10 days post-infection (dpi) and (**C**) 36 dpi in the serum of puppies. The rich factor is represented by the X-axis label, and the KEGG pathway name is displayed on the Y-axis label. The percentage of DEmiRNAs-targeted genes in a specific pathway is reflected in the rich factor. The dots’ colors represent the enrichment score [−log_10_(*p*-value)], with green representing low enrichment and red representing high enrichment. The number of targeted genes of DEmiRNAs in the appropriate pathway is represented by the size of the dots (bigger dots indicate larger number of DEmiRNAs targeted genes). “AGE-RAGE signaling pathway…” was “AGE-RAGE signaling pathway in diabetic complications”.

At 24 hpi, 5722 predicted target genes of the DEmiRNAs were significantly enriched in 91 KEGG pathways, such as protein digestion and absorption, ECM-receptor interaction, amoebiasis and focal adhesion. At 10 dpi, 11,498 predicted target genes of the DEmiRNAs were significantly enriched in 118 KEGG pathways; among which, some pathways were associated with parasitic infectious diseases, such as amoebiasis, toxoplasmosis and chagas disease, as well as the pathways associated with immune system, such as platelet activation, IL-17 signaling pathway and chemokine signaling pathway. At 36 dpi, 9653 predicted target genes of the DEmiRNAs were significantly enriched in 255 KEGG pathways; among which, some pathways were related to infectious diseases, digestive system, cellular community, signaling molecules and interaction, and endocrine and metabolic diseases (Table S3). The top 15 KEGG pathways at each infection stage are shown in Figure 3.

## 4. Discussion

Up to now, although some advances have been achieved in the studies of *T. canis* biology, physiology and pathogenesis [9], the interaction mechanisms between *T. canis* and its definitive canine hosts remain poorly understood. miRNAs can affect the functions of various immune cells, and regulate the various biological processes of the host [15]. Therefore, we analyzed the global miRNA expression profiles in the serum of Beagle dogs infected by *T. canis* at different stages using RNA-seq. In this study, to mimic natural infections as much as possible, we infected puppies with 300 infectious eggs instead of 1000 or more. Therefore, in this case, the puppies did not have an intense pathological response, which is consistent with our expectations, because *T. canis* can evade the immune surveillance of the host through a variety of strategies. When performing the data analysis, we also performed the “FDR value (*p* adjusted)” analysis; however, we found that only a very limited number of DEmiRNAs were screened out when using the “*p* adjusted” analysis. Therefore, we believe that it is reasonable to use “*p*-value” as a screening criterion when clarifying the true circumstances, instead of using *p* adjusted. The expression profiles of hepatic miRNAs were identified in Beagle dogs infected by *T. canis*, with 9, 16 and 34 DEmiRNAs at 12 hpi, 24 hpi and 36 dpi, respectively, which participated in many immune- or inflammation-related signaling pathways [16], suggesting that miRNAs can play critical roles in the pathogenesis of *T. canis* infection. However, at 24 hpi, only three DEmiRNAs were identified in the serum of Beagle dogs in this study, which was much less than the 16 DEmiRNAs detected in Beagle dog livers at the same time [16], suggesting that *T. canis* has a slight effect on the transcription level of miRNAs in the serum of Beagle dogs at 24 hpi.

At 10 dpi, *T. canis* larvae return to the digestive tract and develop into fourth stage larvae (L4). Although the L4 reside in the digestive tract, they can still significantly alter the transcription level of miRNAs in the serum of Beagle dogs, such as cfa-miR-92b, cfa-miR-122 and cfa-miR-485. miR-92b can participate in many diseases, such as lung cancer and diabetic nephropathy [26,27]. miR-92b can perform multiple functions by regulating multiple target mRNAs, such as suppressing viability and invasion by targeting EZH2 in breast cancer [28], and attenuate inflammation by affecting the expression of TRAF3 and inhibiting the MKK3-p38 pathway in acute pancreatitis [29]. In this study, the level of miR-92b was downregulated 3.86 times in the serum of Beagle dogs infected with *T. canis*. However, there is no research on the role of miR-92b in parasite infection, thus it is necessary to further reveal the function of the downregulated miR-92b in host serum after *T. canis* infection. miR-122 also has a wide range of biological functions, such as regulating apoptosis, autophagy, cardiovascular inflammation, dysfunction, fibrosis and oxidative stress [30]. miR-122 is a potential circulating biomarker in some pathogenic infections and it is a central player in liver biology and disease; for example, miR-122 was upregulated in the serum of patients with HBV chronic infection, while it was downregulated in the serum of patients with HCV chronic infection [31]. The expression level of miR-122 was downregulated in mouse liver with visceral leishmaniasis; conversely, restoration of miR-122 levels can reduce the burden of *Leishmania donovani* in liver [32]. The transcription level of miR-122 was not significantly different in dog livers infected with *T. canis* [16]; however, in this study, miR-122 was upregulated 5.69 times in the serum of Beagle dogs at 10 dpi, suggesting that miR-122 may have potential to act as a circulating biomarker in the serum of Beagle dogs at the middle stage of the *T. canis* infection. miR-485 participates in multiple biological processes, such as regulating antiviral immunity and restricting viral replication [33], inhibiting metastasis of lung adenocarcinoma by targeting Flot2 [34], and alleviating epilepsy in cellular and rodent models [35], suggesting that miR-485 plays a positive role in maintaining the body homeostasis, but this phenomenon does not seem to appear in the lung and liver of Beagle dogs infected with *T. canis*, where the expression of miR-485 was not significantly different [11,16]. However, it is worth noting that, in this study, the transcription level of miR-485 was downregulated 2.76 times in dog serum at 10 dpi. Some predicted target genes of DEmiRNAs were involved in the pathways associated with parasitic infectious diseases, such as amoebiasis, toxoplasmosis and chagas disease, as well as pathways associated with immune system, such as platelet activation, IL-17 signaling pathway and chemokine signaling pathway, suggesting that the altered miRNAs induced by *T. canis* infection may be involved in multiple immune processes at 10 dpi.

At 36 dpi, *T. canis* larvae return to the small intestine and develop into adult worms. Different from the downregulation of 3.86 times at 10 dpi, the transcription level of cfa-miR-92b was upregulated 4.54 times at 36 dpi. The difference in miR-92b expression patterns at different stages of *T. canis* infection may have different effects on the development of *T. canis* in definitive hosts. The let-7 gene was originally discovered in *Caenorhabditis elegans*, and then it was identified and studied in many other organisms [36]. The reduction of let-7g expression has been associated with multiple inflammation processes [37]. The downregulation of let-7g was found in the liver infected with *Plasmodium chabaudi* [38], and in the liver infected with *T. canis* [16]. In this study, the transcription level of let-7g was downregulated 3.48 times in dog serum at 36 dpi, suggesting that let-7g could participate in the inflammation process of *T. canis* infection in definitive host. miR-16 is an important miRNA that could be a potential biomarker for human cancer diagnosis [39]. miR-451 is abundant in plasma and highly specific in the erythroid lineage, such as circulating red blood cells [40]. Malaria parasites can reproduce in the red blood cells, and the plasma miR-16 and miR-451 were downregulated in malaria patients infected by *Plasmodium vivax* [41]. In this study, the transcription level of miR-16 and miR-451 were downregulated 4.35 and 3.80 times in dog serum at 36 dpi, suggesting that miR-16 and miR-451 could play important roles in *T. canis* infection. *Clonorchis sinensis* is a carcinogenic human liver fluke [42], and the excretory–secretory products (ESPs) of *C. sinensis* can induce the upregulation of cancer-related miR-93 in ESPs-treated H69 cells [43]. In this study, the transcription level of miR-93 was downregulated 3.66 times in dog serum at 36 dpi. Adult *T. canis* in the gut can affect the host through the ESPs [8,11]. However, little studies were performed to explore the effects of ESPs of *T. canis* on the host/host cells. Moreover, some predicted target genes of the DEmiRNAs were involved in the pathways related to infectious diseases, digestive system, cellular community, signaling molecules and interaction, and endocrine and metabolic diseases.

Although the experimental animals used in this study were the highest standard Beagle dogs available in China, which were purchased from and housed at the National Canine Laboratory Animal Resource Center (Guangzhou, China), only three infected puppies and three control puppies were examined at each of the indicated time points post infection, which is a limitation of the present study. Therefore, to further explore the biological functions of these altered miRNAs in the serum of Beagle dogs infected with *T. canis*, as well as to validate the changes of these DEmiRNAs using qPCR, it would be necessary to use a larger number of Beagle dogs in future studies. Further studies of these DEmiRNAs in dog serum, and their predicted target genes and associated pathways may contribute to the understanding of migration and development of *T. canis* in the definitive host.

## 5. Conclusions

In this study, we revealed altered miRNA expression profiles in the serum of Beagle dogs experimentally infected with *T. canis* at different stages. A total of 3, 25 and 25 DEmiRNAs were identified in dog serum at 24 hpi, 10 dpi and 36 dpi, respectively. These miRNAs are involved in the parasitic infectious diseases- and immune system-related pathways. These results provided a foundation to explore the underlying regulatory role of miRNAs in definitive hosts after *T. canis* infection. Future studies are needed to explore the biological functions of these altered miRNAs in dog serum, which may contribute to the understanding of the regulatory signaling networks involved in the pathogenesis of toxocariasis, and may provide targets for therapeutic interventions.

## Figures and Tables

**Table 1:** Primers used for the quantitative real-time PCR experiment in this study.

Genes	Forward Primers
novel_mir156	GTGAGATGCCAGAGGAAGGTG
novel_mir320	ATCCTAAGGTTGGACGGTCTGG
novel_mir93	CTGGTGAGAACAGCAGGACG
cfa-miR-485	CTAGAAGAGGCTGGCCGTGAT
novel_mir529	CTGGTAGGAAGGGTGGTAGGG
cfa-miR-222	AGCTACATCTGGCTACTGGGT
cfa-miR-128	CACTCACAGTGAACCGGTCTC
cfa-miR-451	CTGGAAACCGTTACCATTACTGAG
cfa-miR-93	CAAAGTGCTGTTCGTGCAGG
cfa-miR-16	GGTCTGGTAGCAGCACGTAA
U6 ^a^	CGCTTCGGCAGCACATATAC

^a^ U6 small nuclear RNA is the house-keeping gene used for normalizing the level of miRNAs.

## Data Availability

All relevant data are within the paper and its supporting information files. The RNA-seq raw data are available in the NCBI SRA repository under accession number PRJNA741736.

## Supplementary Materials

The following supporting information can be downloaded at: https://www.mdpi.com/article/10.3390/ani13020299/s1, Table S1: The total known and predicted miRNAs in Beagle dog serum in this study; Table S2: The differently expressed miRNAs in Beagle dog serum infected by *Toxocara canis* at 24 hpi, 10 dpi and 36 dpi, respectively; Table S3: Signaling pathway enrichments of the differently expressed miRNAs in Beagle dog serum infected by *Toxocara canis* at 24 hpi, 10 dpi and 36 dpi, respectively.
